# Radiation Therapy in Thoracic Tumors: Recent Trends and Current Issues

**DOI:** 10.3390/cancers14112706

**Published:** 2022-05-30

**Authors:** Laura Cella, Giuseppe Palma

**Affiliations:** 1 Institute of Biostructures and Bioimaging, National Research Council, 80145 Naples, Italy; 2 Institute of Nanotechnology, National Research Council, 73100 Lecce, Italy

Radiation therapy (RT) plays a fundamental role in the multidisciplinary treatment and management of thoracic cancers, and in particular, RT is the most used non-surgical treatment modality for lung cancer, which in turn is the most common type of thoracic malignancy [1]. Thanks to the recent technological progress in modern RT techniques and new fractionation paradigms, an improved therapeutic ratio has led to better long-term disease control and reduced radiation-related side effects [2].

This Special Issue analyzes the multifaceted aspects of radiation therapy in thoracic tumors and highlights potential further improvements in treatment strategies to tailor thoracic cancer care.

High-precision RT techniques, such as intensity-modulated RT (IMRT), particle therapy or stereotactic body RT (SBRT), are required for improved imaging to account for tumor motion and uncertainties. Four-dimensional computed tomography (CT) is now routinely used in planning thoracic RT to create target volumes based upon the motion of the tumor during the imaging study [3]. An alternative strategy to account for organ motion is respiratory gating, where the treatment beam is only switched on when the tumor is in a specific location [4]. In this framework, Kraus et al. [5] investigated the potential of respiratory gating to mitigate motion-caused misdosage in lung SBRT. A normal-tissue complication probability (NTCP) model analysis showed a sensible reduction in pulmonary and esophageal toxicity for moderate gating window sizes. Accordingly, the authors suggest a pretreatment toxicity risk analysis to facilitate efficient patient selection for gating and the choice of the optimal gating window.

The increasing complexity of the parameters’ space characteristic of modern RT techniques also fosters an automated approach to treatment planning optimization in order to improve plan quality while reducing hands-on planning time [6]. An example of an automated multi-criterial treatment planning system (TPS) is the Erasmus-iCycle with integrated beam angle optimization, developed at the Erasmus University Medical Center. In [7], the Erasmus-iCycle was coupled with the commercial TPS Eclipse (Varian Medical Systems, Palo Alto, CA, USA), obtaining the novel “iCE” system. Its potential was shown in Locally Advanced Non-Small-Cell Lung Cancer (NSCLC) patients, where an improved sparing of the heart and esophagus was observed for most of the analyzed patients, with significant reductions in heart and esophagus dosimetric parameters predictive for toxicity.

Among the new techniques, SBRT represents a relevant therapeutic treatment option in inoperable patients (or those refusing surgery) with early-stage NSCLC or lung oligometastases. It is usually administered in 3 to 10 fractions, while the adoption of single-fraction SBRT is instead still limited [8]. The results from the systematic review of nine retrospective trials by Bartl et al. [9] suggest single-fraction SBRT as an efficacious and well-tolerated definitive treatment, with local control rates of over 90%, favorable survival measures, and mild toxicity profiles.

For inoperable locally advanced (LA)-NSCLC patients, the standard of care is IMRT with concurrent chemotherapy. This approach is preferred over sequential chemo-RT and RT alone due to better survival. A real-world patient population analysis studied the relationship between dosimetric parameters, overall survival and toxicity in patients with stage III NSCLC treated with IMRT/VMAT and/or chemotherapy [10].

For LA-NSCLC patients, proton therapy (PT) has been suggested as a viable option to escalate dose prescription while reducing side effects [11]. In particular, intensity-modulated PT (IMPT) has the potential to improve the conformality of the dose distributions when compared with conventional photon plans [12]. However, IMPT is also more affected by the influence of uncertainties due to breathing and anatomical changes, as argued by Boer et al. [13]. In their prospective simulation study, the influence of such uncertainties on treatment delivery was thoroughly investigated.

Recently, the use of PT has also been evaluated for the treatment of mediastinal Hodgkin Lymphoma (HL) due to its potential to reduce the dose given to organs-at-risk, and in particular, to cardiac substructures [14]. However, clinical evidence for this technique in mediastinal HL irradiation is still limited. An interesting analysis of the available clinical data from published HL proton therapy studies is provided in [14]. Multiple issues hamper the democratization of HL proton therapy, as highlighted in [15], where current challenges and controversies that may impede the larger-scale implementation of mediastinal HL proton therapy were reviewed.

Thoracic RT is often associated with the risk of developing acute or late radiation-induced morbidities for which robust toxicity prediction models are required [16]. In [17], the prognostic factors for radiation-induced dyspnea after SBRT for NSCLC were investigated, and a logistic predictive model including clinical and dosimetric variables was proposed.

As for radiation-induced esophagitis and lung damage after thoracic RT, a wide range of conflicting dosimetric factors have been reported as toxicity predictors, thus highlighting the potential limits of the performed analyses. Indeed, for both organs, an inhomogeneous radiosensitivity has been hypothesized, and a new method for investigating such organ characteristics has been proposed (the so-called voxel-based analysis—VBA [18]). In [19], Monti et al. analyzed the dose patterns associated with esophagitis via different VBAs, and NTCP models with good prediction performance were finally developed. As two of the limiting factors of VBA in radiation oncology are the spatial dose autocorrelation and the inhomogeneity of the voxelwise probability density function of the dose, in [20] several tools for a preliminary assessment of dose distributions are proposed. The authors include a probabilistic independent component analysis of the dose maps and a connectogram representation of their autocorrelations.

The training of NTCP models relies on the classification of patients according to the development of a given toxicity. In this context, the papers by Chandy et al. [21] and Szmul et al. [22] present two automated classification algorithms for the analysis of RT-induced lung damage in NSCLC patients, providing novel insights into the temporal evolution of lung damage and its relationship with global and local dose as well as respiratory outcomes [21].

This Special Issue offers significant viewpoints on several current hot topics in the RT of thoracic malignancies, and the published papers are likely to stimulate valuable improvements in cancer treatment.
